# Crystal structures and Hirshfeld surface analyses of 2-[(4,6-di­amino­pyrimidin-2-yl)sulfan­yl]-*N*-(pyridin-2-yl)acetamide and 2-[(4,6-di­amino­pyrimidin-2-yl)sulfan­yl]-*N*-(pyrazin-2-yl)acetamide

**DOI:** 10.1107/S2056989018005704

**Published:** 2018-04-27

**Authors:** Manisha Choudhury, Vijayan Viswanathan, Ajay Kumar Timiri, Barij Nayan Sinha, Venkatesan Jayaprakash, Devadasan Velmurugan

**Affiliations:** a Centre of Advanced Study in Crystallography and Biophysics, University of Madras, Guindy Campus, Chennai 600 025, India; b Department of Pharmaceutical Science and Technology, Birla Institute of Technology, Mesta, Ranchi 835215, Jharkhand, India

**Keywords:** crystal structure, 4,6-di­amino­pyrimidine, sulfan­yl, acetamide, pyridine, pyrazine, hydrogen bonding, offset π-π inter­actions, Hirshfeld surface

## Abstract

In the title compounds, C_11_H_12_N_6_OS (I) and C_10_H_11_N_7_OS (II), the di­amino­pyrimidine ring makes dihedral angles of 71.10 (9)° with the pyridine ring in (I) and 62.93 (15)° with the pyrazine ring in (II). The ethanamine group, –CH_2_–C(=O)–NH– lies in the plane of the pyridine and pyrazine rings in compounds (I) and (II), respectively. In both compounds, there is an intra­molecular N—H⋯N hydrogen bond forming an *S*(7) ring motif and a short C—H⋯O inter­action forming an *S*(6) loop. In the crystals of both compounds, mol­ecules are linked by pairs of N—H⋯N hydrogen bonds, forming inversion dimers with *R*_2_^2^(8) ring motifs. In (I), the dimers are linked by N—H⋯O and N—H⋯N hydrogen bonds, forming layers parallel to (1 ). The layers are linked by offset π–π inter­actions [inter­centroid distance = 3.777 (1) Å], forming a three-dimensional supra­molecular structure. In (II), the dimers are linked by N—H⋯O, N—H⋯N and C—H⋯O hydrogen bonds, also forming a three-dimensional supra­molecular structure.

## Abstract 2

The conformation of the title di­amino­pyrimidine sulfanly acetamides, (I) and (II), have similar conformations, with the pyrimidine ring being inclined to the pyridine ring in (I) by 71.10 (9) °, and by 62.93 (15) ° to the pyrazine ring in (II).

## Chemical context   

An important property of di­amino­pyrimidine derivatives is their inhibition potential against cancer targets. Because of the limited capacity of drugs that can cure cancer, there is always an urgent requirement for new chemotherapeutics. 2,4-Di­amino­pyrimidine derivatives combined with aryl­thia­zole derivatives have shown to possess significant anti-proliferation properties against breast cancer cell lines (Zhou *et al.*, 2015 ▸). 2,4-Di­amino­pyrimidine derivatives have shown significant inhibitory activity against influenza viruses (Kimura *et al.*, 2006 ▸). A series of 2,4- di­amino­pyrimidine derivatives were evaluated against Bacillus anthracis, which showed inhibition (Nammalwar *et al.*, 2012 ▸). Di­hydro­folate reductase inhibitor drugs such as pyrimethamine, trimetrexate and piritrexim (Nelson & Rosowsky, 2001 ▸) and the anti­biotics iclaprim and trimethoprim all include di­amino­pyrimidine as the fundamental structural motif. Di­amino­pyrimidine derivatives have also shown anti-retroviral activity (Hocková *et al.*, 2004 ▸), anti­bacterial (Kandeel *et al.*, 1994 ▸) and potential anti-microbial properties (Holla *et al.*, 2006 ▸). As part of our own studies in this area, we report herein on the syntheses, crystal structures and Hirshfeld surface analyses of the title compounds, 2-[(4,6-di­amino­pyrimidin-2-yl)sulfan­yl]-*N*-(pyridin-2-yl)acet­amide (I) and 2-[(4,6-di­amino­pyrimidin-2-yl)sulfan­yl]-*N*-(pyrazin-2-yl)acetamide (II).
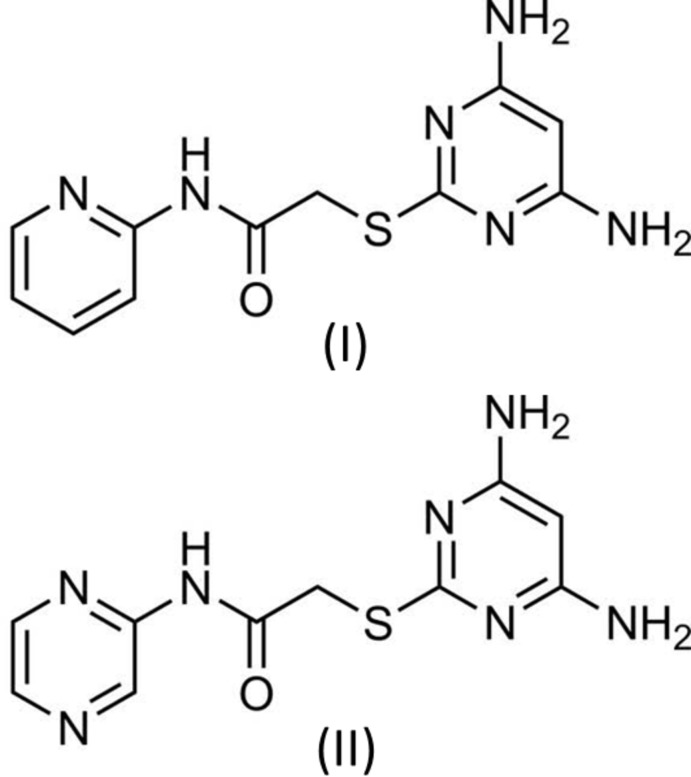


## Structural commentary   

**Figure 1:**
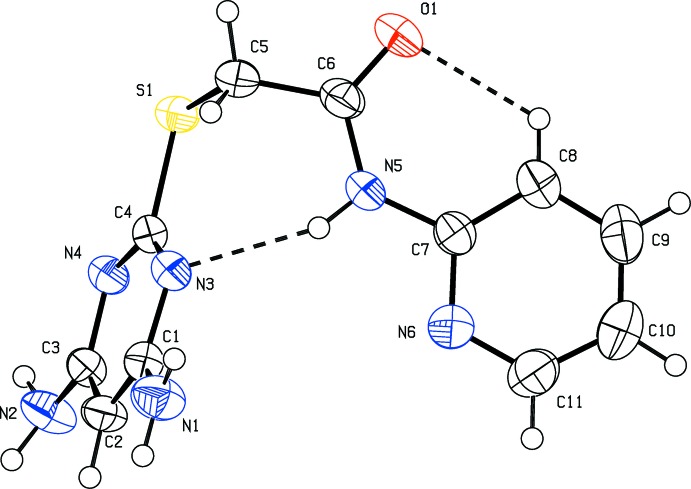
The mol­ecular structure of the compound (I), showing the atom labelling and displacement ellipsoids drawn at the 50% probability level. The intra­molecular N—H⋯N and C—H⋯O hydrogen bonds (see Table 1 ▸) are shown as dashed lines.

The mol­ecular structure of compounds (I) and (II) are shown in the Figs. 1 ▸ and 2 ▸, respectively. Compound (I) crystallizes in the triclinic space group *P* 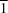 and compound (II) crystallizes in the monoclinic space group *P*2_1_/*c*. In both the compounds, there is an intra­molecular N—H⋯N hydrogen bond forming an *S*(7) ring motif and a short C—H⋯O inter­action forming an *S*(6) loop; see Tables 1 ▸ and 2 ▸ for details of the hydrogen bonding. The nitro­gen atoms N1 and N2 lie in the plane of the pyrimidine ring to which they are attached [deviations are −0.0269 (17) and 0.0521 (16) Å, respectively, for compound (I), and 0.0350 (28) and 0.0284 (28) Å, respectively, for compound (II)]. The di­amino­pyrimidine ring makes a dihedral angle of 71.10 (9)° with the pyridine ring in compound (I) and a dihedral angle of 62.93 (15)° with the pyrazine ring in compound (II). In (I) the ethanamine group (N5/O1/C6/C5) and the pyridine ring are coplanar, as evidenced by torsion angle C7—N5—C6—C5 = 179.1 (2)°. In (II) the ethanamine group (N5/O1/C6/C5) and pyrazine ring also lie in a plane [C7—N5—C6—C5 = 177.6 (3)°]. Bond lengths C4—S1 [1.768 (2) Å] and C5—S1 [1.802 (2) Å] for compound (I), and C4—S1 [1.768 (3) Å] and C5—S1 [1.795 (3) Å] for compound (II), are comparable with values reported for similar compounds (see Section 4. *Database survey*).

## Supra­molecular features   

**Figure 3:**
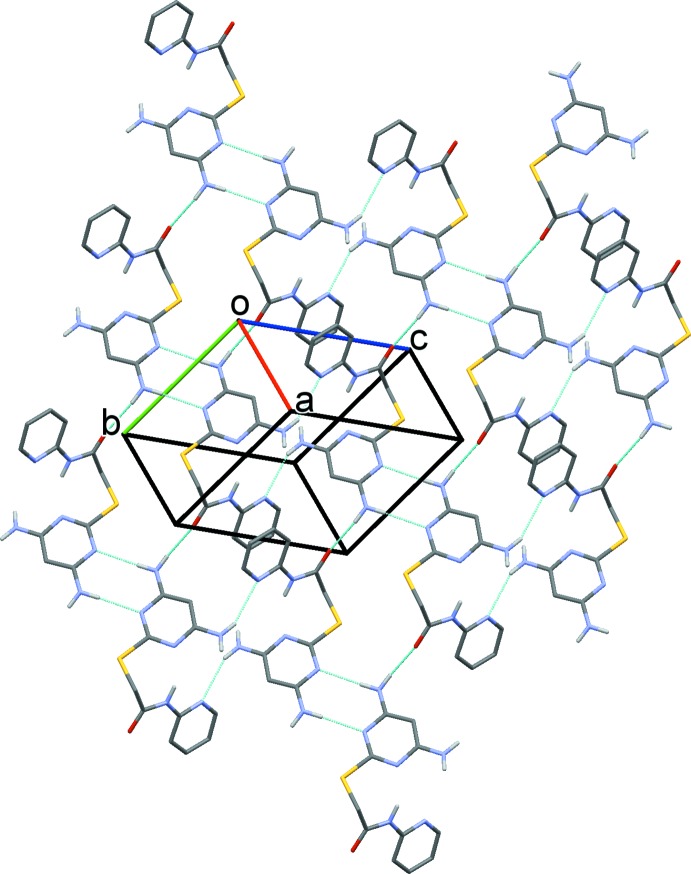
A view normal to the (1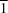 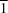) plane of the crystal packing of compound (I). The hydrogen bonds (see Table 1 ▸) are shown as dashed lines and C-bound H atoms have been omitted for clarity.

**Figure 4:**
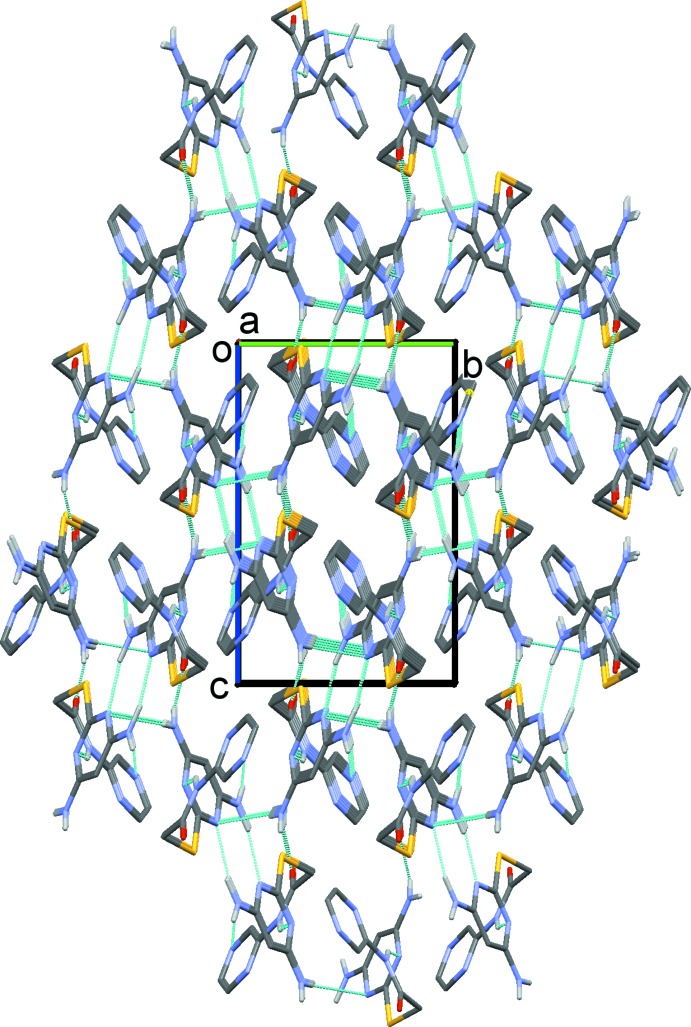
A view along the *a* axis of the crystal packing of compound (II). The hydrogen bonds (see Table 2 ▸) are shown as dashed lines, and C-bound H atoms have been omitted for clarity.

The crystal packing in compound (I) is illustrated in Fig. 3 ▸, and that for compound (II) in Fig. 4 ▸. Details of the hydrogen-bonding geometry in compound (I) are given in Table 1 ▸ and in Table 2 ▸ for (II). In the crystals of both compounds, mol­ecules are linked by pairs of N2—H2*B*⋯N4^i^ hydrogen bonds, forming inversion dimers with 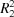
(8) ring motifs (Figs. 3 ▸ and 4 ▸, respectively).

In the crystal of (I), the dimers are linked by N2—H2*A*⋯O1^iii^ hydrogen bonds, forming ribbons along [010], enclosing 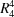
(18) ring motifs. Adjacent ribbons are linked by N1—H1*A*⋯N6^ii^ hydrogen bonds, forming sheets lying parallel to the (1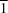 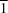) plane, see Fig. 3 ▸. The layers are linked by offset π–π inter­actions, forming a three-dimensional supra­molecular structure [*Cg*⋯*Cg* ^v^ = 3.777 (1) Å, inter­planar distance = 3.483 (1) Å, slippage = 1.459 Å, *Cg* is the centroid of the pyridine ring (N6/C7–C11); symmetry code: (v) −*x* + 1, −*y*, −*z* + 1)].

In the crystal of (II), the dimers are linked by N1—H1*A*⋯O^ii^, N2—H2*A*⋯N7^iii^ and C9—H9⋯O1^iv^ hydrogen bonds (Table 2 ▸), forming a three-dimensional supra­molecular structure (Fig. 4 ▸). In contrast, in the crystal of (II) there are no π–π inter­actions present.

## Database survey   

A search of the Cambridge Structure Database (Version 5.39, last update February 2018; Groom *et al.*, 2016 ▸) for [(4,6-di­amino­pyrmidin-2-yl)sulfan­yl]acetamide yielded nine hits, eight of which have a substituted phenyl substituent in place of the pyridine ring in (I) and the pyrazine ring in (II), and one a naphthalene group (JARPOK; Subasri *et al.*, 2017*a* ▸). They include the following analogues: 3-nitro­phenyl (ARAROC; Subasri *et al.*, 2016 ▸), 2-chloro­phenyl (ARARUI; Subasri *et al.*, 2016 ▸), 2-methyl­phenyl (GOKWIO; Subasri *et al.*, 2014 ▸), 4-fluoro­phenyl (JARPUQ; Subasri *et al.*, 2017*a* ▸), 2,4-di­methyl­phenyl (JAXFIA; Choudhury *et al.*, 2017 ▸), 3-meth­oxy­phenyl (JAXFOG; Choudhury *et al.*, 2017 ▸), 4-chloro­phenyl (KAPQIE; Subasri *et al.*, 2017*b* ▸), and 3-chloro­phenyl (KAPQOK; Subasri *et al.*, 2017*b* ▸).

In these eight compounds, the di­amino­pyrimidine and benzene rings are inclined to one another by dihedral angles varying from *ca* 42.25 to 78.33°. The dihedral angle between the di­amino­pyrimidine and the pyridine ring in (I) is 71.10 (9)° and with the pyrazine ring in (II) is 62.93 (15)°, well within these limits. As in the title compounds, there is also an intra­molecular N—H⋯N hydrogen bond present in all eight compounds, stabilizing the folded conformation of each mol­ecule. In the crystals of all but two compounds (ARAROC and JARPUQ), mol­ecules are linked by pairs of N—H⋯N hydrogen bonds, involving the 4,6-di­amino­pyrimidine moieties, forming inversion dimers with 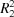
(8) ring motifs, as for compounds (I) and (II).

## Hirshfeld surface analysis   

**Figure 5:**
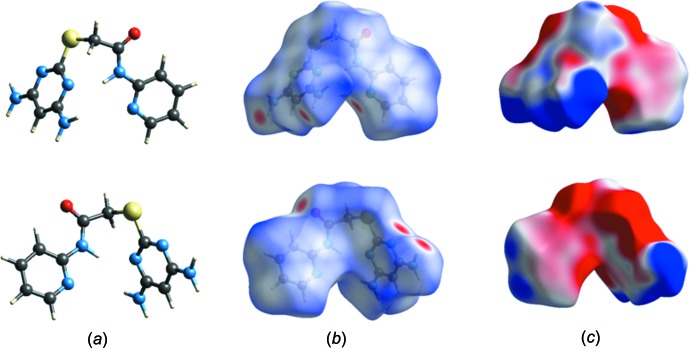
Ball and stick, Hirshfeld surface and electrostatic potential surface diagrams for compound (I).

**Figure 7:**
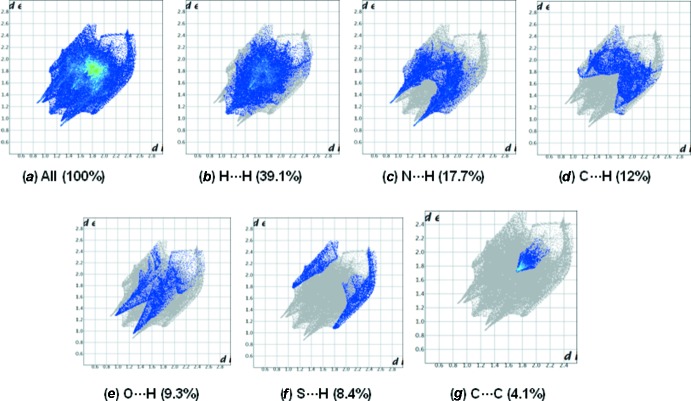
The 2D fingerprint plot for all the inter­molecular contacts for compound (I).

**Figure 8:**
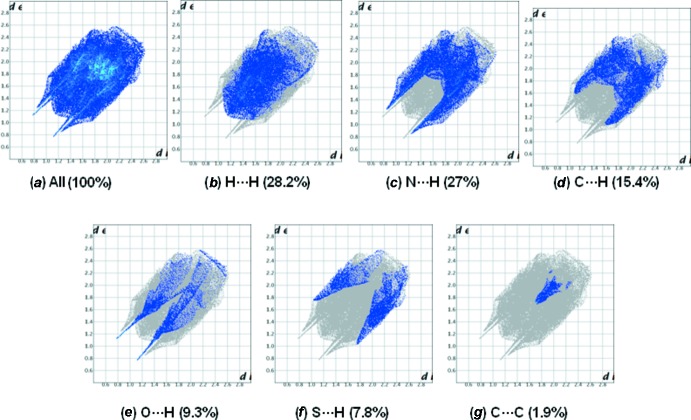
The 2D fingerprint plot for all the inter­molecular contacts for compound (II).

In Figs. 5 ▸ and 6 ▸, the ball and stick model of the front and back views of the compounds (I) and (II), respectively, and the inter­molecular contacts are shown by conventional mapping of *d*_norm_ on the mol­ecular Hirshfeld surfaces, where the red-spot areas denote inter­molecular contacts involved in the hydrogen-bonding inter­actions (McKinnon *et al.*, 2007 ▸). The electrostatic potential is mapped on the Hirshfeld surface using the STO-3G basis set at the Hartree–Fock theory over the range of ±0.025 a.u. The positive electrostatic potential (blue region) over the surface shows hydrogen-donor potential, and the hydrogen-bond acceptors are shown by negative electrostatic potential (red regions); see Figs. 5 ▸ and 6 ▸. The two-dimensional fingerprint plots [Fig. 7 ▸ for (I) and Fig. 8 ▸ for (II)] are deconvoluted to highlight atom-pair close contacts by which different atomic types, overlapping the full fingerprint can be separated based on different inter­action types. For compound (I), inter­molecular H⋯H contacts of 39.1% are the most significant, followed by 17.7% for N⋯H/H⋯N, 12% for C⋯H/H⋯C, 9.3% for O⋯H/H⋯O, 8.4% for S⋯H/H⋯S and 4.1% for C⋯C contacts. In contrast, for compound (II) the H⋯H contacts at 28.2% are significantly lower than in (I), while the N⋯H/H⋯N contacts at 27% are significantly higher than in (I). The C⋯C contacts at only 1.9% are much lower than in (I) where offset π–π inter­actions are observed in the crystal structure.

## Synthesis and crystallization   

**Compound (I):** To a solution of 4, 6-di­amino-pyrimidine-2-thiol (0.5 g; 3.52 mmol) in 25 ml of ethanol, (0.2g; 3.52 mmol) potassium hydroxide was added and refluxed for about 30 min. Then an equimolar qu­antity of 2-chloro-*N*-(pyridin-2-yl)acetamide (3.52 mmol) was added to the above reaction mixture and it was refluxed for 5 h. Evaporation of the organic layer under vacuum provided compound (I). After purification, the compound was crystallized from ethanol solution by slow evaporation of the solvent giving yellow block-like crystals.

**Compound (II)**: To a solution of 4, 6-di­amino-pyrimidine-2-thiol (0.5 g; 3.52 mmol) in 25 ml of ethanol, (0.2g; 3.52 mmol) potassium hydroxide was added and refluxed for about 30 min. Then an equimolar qu­antity of 2-chloro-*N*-(pyrazin-2-yl)acetamide (3.52 mmol) was added to the above reaction mixture and it was refluxed for 5.5 h. Evaporation of the organic layer under vacuum resulted in compound (II). After purification, the compound was crystallized from ethanol solution by slow evaporation of the solvent giving yellow block-like crystals.

## Refinement   

Crystal data, data collection and structure refinement details are summarized in Table 3 ▸. For both compounds, the NH_2_ and NH H atoms were located in difference-Fourier maps and freely refined, and the C-bound H atoms were placed in calculated positions and refined in the riding model: C—H = 0.93–0.97 Å with *U*_iso_(H) = 1.2*U*_eq_(C).

## Supplementary Material

Crystal structure: contains datablock(s) global, I, II. DOI: 10.1107/S2056989018005704/su5430sup1.cif

Structure factors: contains datablock(s) I. DOI: 10.1107/S2056989018005704/su5430Isup4.hkl

Structure factors: contains datablock(s) II. DOI: 10.1107/S2056989018005704/su5430IIsup5.hkl

Supporting information file. DOI: 10.1107/S2056989018005704/su5430Isup4.cml

Supporting information file. DOI: 10.1107/S2056989018005704/su5430IIsup5.cml

CCDC references: 1836419, 1836418

Additional supporting information:  crystallographic information; 3D view; checkCIF report

## Figures and Tables

**Figure 2:**
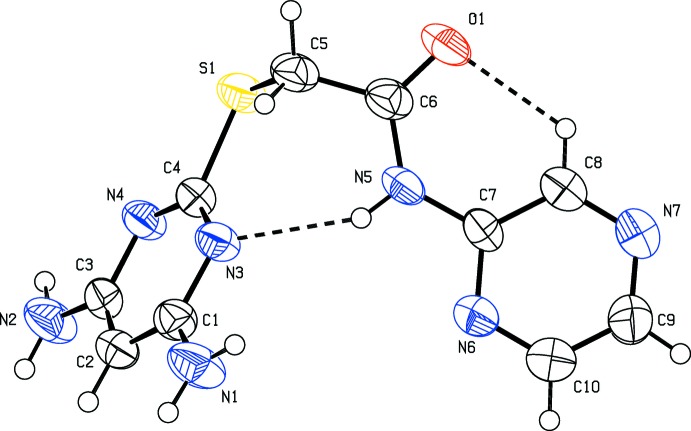
The mol­ecular structure of the compound (II), showing the atom labelling and displacement ellipsoids drawn at the 50% probability level. The intra­molecular N—H⋯N and C—H⋯O hydrogen bonds (see Table 2 ▸) are shown as dashed lines.

**Figure 6:**
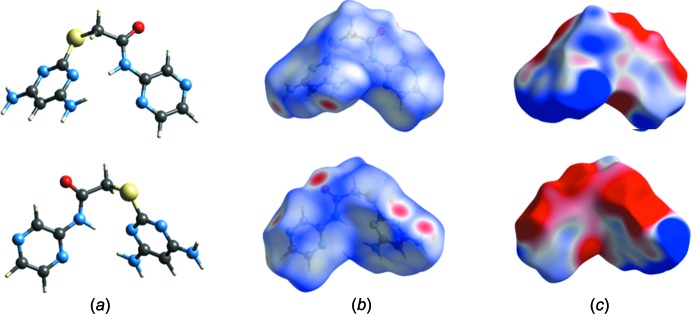
Ball and stick, Hirshfeld surface and electrostatic potential surface diagrams for compound (II).

**Table 1:** Hydrogen-bond geometry (Å, °) for (I)

*D*—H⋯*A*	*D*—H	H⋯*A*	*D*⋯*A*	*D*—H⋯*A*
N5—H5⋯N3	0.86 (2)	2.18 (2)	2.975 (2)	154 (2)
C8—H8⋯O1	0.93	2.31	2.894 (2)	121
N2—H2*B*⋯N4^i^	0.88 (2)	2.20 (2)	3.082 (2)	178 (2)
N1—H1*A*⋯N6^ii^	0.86 (2)	2.38 (2)	3.174 (2)	155 (2)
N2—H2*A*⋯O1^iii^	0.86 (2)	2.13 (2)	2.956 (2)	159 (2)

Symmetry codes: (i) 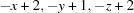; (ii) 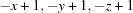; (iii) 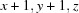.

**Table 2:** Hydrogen-bond geometry (Å, °) for (II)

*D*—H⋯*A*	*D*—H	H⋯*A*	*D*⋯*A*	*D*—H⋯*A*
N5—H5⋯N3	0.82 (3)	2.25 (3)	2.993 (4)	151 (3)
C8—H8⋯O1	0.93	2.24	2.854 (4)	123
N2—H2*B*⋯N4^i^	1.00 (3)	2.11 (3)	3.092 (4)	169 (3)
N1—H1*A*⋯O1^ii^	0.86 (3)	2.06 (4)	2.904 (4)	167 (3)
N2—H2*A*⋯N7^iii^	0.85 (3)	2.41 (3)	3.235 (4)	164 (3)
C9—H9⋯O1^iv^	0.93	2.56	3.368 (4)	145

Symmetry codes: (i) 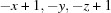; (ii) 
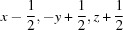
; (iii) 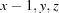; (iv) 
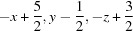
.

**Table 3:** Experimental details

	(I)	(II)
Crystal data		
Chemical formula	C_11_H_12_N_6_OS	C_10_H_11_N_7_OS
*M*_r_	276.33	277.32
Crystal system, space group	Triclinic, *P* 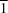	Monoclinic, *P*2_1_/*n*
Temperature (K)	293	293
*a*, *b*, *c* (Å)	7.2341 (2), 9.3852 (2), 9.7971 (2)	12.1333 (5), 8.1561 (3), 12.8442 (5)
α, β, γ (°)	95.820 (1), 91.116 (1), 105.682 (1)	90, 94.307 (3), 90
*V* (Å^3^)	636.33 (3)	1267.48 (9)
*Z*	2	4
Radiation type	Mo *K*α	Mo *K*α
μ (mm^−1^)	0.26	0.26
Crystal size (mm)	0.30 × 0.25 × 0.20	0.28 × 0.25 × 0.20

Data collection		
Diffractometer	Bruker SMART APEXII area-detector	Bruker SMART APEXII area-detector
Absorption correction	Multi-scan (*SADABS*; Bruker, 2008 ▸)	Multi-scan (*SADABS*; Bruker, 2008 ▸)
*T*_min_, *T*_max_	0.742, 0.841	0.723, 0.863
No. of measured, independent and observed [*I* > 2σ(*I*)] reflections	9447, 2605, 2160	11968, 3124, 1320
*R*_int_	0.020	0.084
(sin θ/λ)_max_ (Å^−1^)	0.626	0.667

Refinement		
*R*[*F* ^2^ > 2σ(*F* ^2^)], *wR*(*F* ^2^), *S*	0.034, 0.094, 1.05	0.054, 0.126, 0.94
No. of reflections	2605	3124
No. of parameters	192	192
H-atom treatment	H atoms treated by a mixture of independent and constrained refinement	H atoms treated by a mixture of independent and constrained refinement
Δρ_max_, Δρ_min_ (e Å^−3^)	0.23, −0.20	0.20, −0.23

Computer programs: *APEX2* and *SAINT* (Bruker, 2008 ▸), *SHELXS97* (Sheldrick, 2008 ▸), *SHELXL2016* (Sheldrick, 2015 ▸), *PLATON* (Spek, 2009 ▸) and *Mercury* (Macrae *et al.*, 2008 ▸).

## supplementary crystallographic information

### 2-[(4,6-Diaminopyrimidin-2-yl)sulfanyl]-N-(pyridin-2-yl)acetamide (I) Crystal data

**Table:**

C_11_H_12_N_6_OS	*Z* = 2
*M**_r_* = 276.33	*F*(000) = 288
Triclinic, *P*1	*D*_x_ = 1.442 Mg m^−^^3^
*a* = 7.2341 (2) Å	Mo *K*α radiation, λ = 0.71073 Å
*b* = 9.3852 (2) Å	Cell parameters from 2605 reflections
*c* = 9.7971 (2) Å	θ = 2.1–26.4°
α = 95.820 (1)°	µ = 0.26 mm^−^^1^
β = 91.116 (1)°	*T* = 293 K
γ = 105.682 (1)°	Block, yellow
*V* = 636.33 (3) Å^3^	0.30 × 0.25 × 0.20 mm

### 2-[(4,6-Diaminopyrimidin-2-yl)sulfanyl]-N-(pyridin-2-yl)acetamide (I) Data collection

**Table:**

Bruker SMART APEXII area-detector diffractometer	2160 reflections with *I* > 2σ(*I*)
Radiation source: X-ray	*R*_int_ = 0.020
ω and φ scans	θ_max_ = 26.4°, θ_min_ = 2.1°
Absorption correction: multi-scan (*SADABS*; Bruker, 2008)	*h* = −9→9
*T*_min_ = 0.742, *T*_max_ = 0.841	*k* = −11→11
9447 measured reflections	*l* = −10→12
2605 independent reflections	

### 2-[(4,6-Diaminopyrimidin-2-yl)sulfanyl]-N-(pyridin-2-yl)acetamide (I) Refinement

**Table:**

Refinement on *F*^2^	Primary atom site location: structure-invariant direct methods
Least-squares matrix: full	Secondary atom site location: difference Fourier map
*R*[*F*^2^ > 2σ(*F*^2^)] = 0.034	Hydrogen site location: mixed
*wR*(*F*^2^) = 0.094	H atoms treated by a mixture of independent and constrained refinement
*S* = 1.05	*w* = 1/[σ^2^(*F*_o_^2^) + (0.0443*P*)^2^ + 0.1669*P*] where *P* = (*F*_o_^2^ + 2*F*_c_^2^)/3
2605 reflections	(Δ/σ)_max_ < 0.001
192 parameters	Δρ_max_ = 0.23 e Å^−^^3^
0 restraints	Δρ_min_ = −0.20 e Å^−^^3^

### 2-[(4,6-Diaminopyrimidin-2-yl)sulfanyl]-N-(pyridin-2-yl)acetamide (I) Special details

**Table:**

Geometry. All esds (except the esd in the dihedral angle between two l.s. planes) are estimated using the full covariance matrix. The cell esds are taken into account individually in the estimation of esds in distances, angles and torsion angles; correlations between esds in cell parameters are only used when they are defined by crystal symmetry. An approximate (isotropic) treatment of cell esds is used for estimating esds involving l.s. planes.

### 2-[(4,6-Diaminopyrimidin-2-yl)sulfanyl]-N-(pyridin-2-yl)acetamide (I) Fractional atomic coordinates and isotropic or equivalent isotropic displacement parameters (Å^2^)

**Table:**

	*x*	*y*	*z*	*U*_iso_*/*U*_eq_	
S1	0.47564 (6)	0.24187 (5)	0.97878 (4)	0.04443 (15)	
O1	0.1201 (2)	−0.07191 (14)	0.81504 (14)	0.0637 (4)	
N1	0.4212 (3)	0.58093 (19)	0.63223 (17)	0.0509 (4)	
H1A	0.470 (3)	0.652 (2)	0.584 (2)	0.059 (6)*	
H1B	0.299 (3)	0.546 (2)	0.641 (2)	0.066 (7)*	
N2	1.0373 (2)	0.60541 (19)	0.83751 (19)	0.0500 (4)	
H2A	1.087 (3)	0.696 (2)	0.820 (2)	0.059 (6)*	
H2B	1.092 (3)	0.590 (2)	0.914 (2)	0.071 (7)*	
N3	0.45530 (19)	0.42030 (14)	0.78589 (14)	0.0378 (3)	
N4	0.75997 (18)	0.44133 (14)	0.89781 (13)	0.0374 (3)	
N5	0.2555 (2)	0.10689 (16)	0.67916 (14)	0.0395 (3)	
H5	0.304 (3)	0.201 (2)	0.6830 (18)	0.046 (5)*	
N6	0.3287 (2)	0.11118 (16)	0.45436 (15)	0.0458 (4)	
C1	0.5404 (2)	0.53494 (17)	0.71413 (16)	0.0373 (4)	
C2	0.7366 (2)	0.59919 (18)	0.72509 (17)	0.0398 (4)	
H2	0.795008	0.672763	0.670694	0.048*	
C3	0.8435 (2)	0.55043 (16)	0.81970 (16)	0.0367 (4)	
C4	0.5717 (2)	0.38421 (16)	0.87394 (15)	0.0351 (4)	
C5	0.2265 (2)	0.17793 (19)	0.91965 (17)	0.0452 (4)	
H5A	0.149123	0.139634	0.994486	0.054*	
H5B	0.185101	0.261192	0.891905	0.054*	
C6	0.1943 (2)	0.05740 (18)	0.80001 (18)	0.0415 (4)	
C7	0.2500 (2)	0.02603 (17)	0.55029 (17)	0.0369 (4)	
C8	0.1697 (3)	−0.12615 (19)	0.5237 (2)	0.0506 (4)	
H8	0.118824	−0.182978	0.593511	0.061*	
C9	0.1674 (3)	−0.1909 (2)	0.3911 (2)	0.0603 (5)	
H9	0.113148	−0.292746	0.369861	0.072*	
C10	0.2447 (3)	−0.1056 (2)	0.2905 (2)	0.0574 (5)	
H10	0.243138	−0.147400	0.200125	0.069*	
C11	0.3246 (3)	0.0435 (2)	0.32694 (19)	0.0551 (5)	
H11	0.379420	0.101358	0.258857	0.066*	

### 2-[(4,6-Diaminopyrimidin-2-yl)sulfanyl]-N-(pyridin-2-yl)acetamide (I) Atomic displacement parameters (Å^2^)

**Table:**

	*U*^11^	*U*^22^	*U*^33^	*U*^12^	*U*^13^	*U*^23^
S1	0.0510 (3)	0.0393 (2)	0.0344 (2)	−0.00414 (19)	0.00358 (18)	0.00918 (17)
O1	0.0848 (10)	0.0348 (7)	0.0585 (8)	−0.0084 (6)	0.0102 (7)	0.0109 (6)
N1	0.0466 (10)	0.0522 (10)	0.0532 (10)	0.0079 (8)	−0.0012 (8)	0.0183 (8)
N2	0.0377 (8)	0.0444 (9)	0.0648 (11)	0.0003 (7)	0.0006 (7)	0.0222 (8)
N3	0.0399 (7)	0.0308 (7)	0.0382 (7)	0.0019 (6)	0.0022 (6)	0.0041 (5)
N4	0.0404 (8)	0.0310 (7)	0.0369 (7)	0.0020 (6)	0.0018 (6)	0.0064 (5)
N5	0.0438 (8)	0.0288 (7)	0.0397 (8)	−0.0008 (6)	0.0032 (6)	0.0044 (6)
N6	0.0523 (9)	0.0425 (8)	0.0418 (8)	0.0106 (7)	0.0039 (7)	0.0070 (6)
C1	0.0443 (9)	0.0331 (8)	0.0332 (8)	0.0084 (7)	0.0034 (7)	0.0030 (6)
C2	0.0427 (9)	0.0349 (8)	0.0411 (9)	0.0058 (7)	0.0085 (7)	0.0127 (7)
C3	0.0395 (9)	0.0281 (8)	0.0397 (9)	0.0042 (6)	0.0063 (7)	0.0040 (6)
C4	0.0427 (9)	0.0266 (7)	0.0311 (8)	0.0022 (6)	0.0056 (7)	−0.0004 (6)
C5	0.0451 (10)	0.0417 (9)	0.0412 (9)	−0.0018 (7)	0.0134 (8)	0.0050 (7)
C6	0.0385 (9)	0.0354 (9)	0.0458 (10)	0.0003 (7)	0.0037 (7)	0.0087 (7)
C7	0.0319 (8)	0.0358 (8)	0.0419 (9)	0.0079 (6)	−0.0010 (7)	0.0038 (7)
C8	0.0531 (11)	0.0375 (9)	0.0546 (11)	0.0026 (8)	0.0031 (9)	0.0009 (8)
C9	0.0629 (13)	0.0441 (11)	0.0672 (13)	0.0099 (9)	−0.0011 (10)	−0.0111 (9)
C10	0.0611 (12)	0.0643 (13)	0.0488 (11)	0.0266 (10)	−0.0007 (9)	−0.0100 (10)
C11	0.0636 (12)	0.0607 (12)	0.0436 (10)	0.0204 (10)	0.0081 (9)	0.0073 (9)

### 2-[(4,6-Diaminopyrimidin-2-yl)sulfanyl]-N-(pyridin-2-yl)acetamide (I) Geometric parameters (Å, º)

**Table:**

S1—C4	1.7682 (15)	N6—C7	1.332 (2)
S1—C5	1.8021 (18)	N6—C11	1.338 (2)
O1—C6	1.2124 (19)	C1—C2	1.381 (2)
N1—C1	1.348 (2)	C2—C3	1.384 (2)
N1—H1A	0.86 (2)	C2—H2	0.9300
N1—H1B	0.86 (2)	C5—C6	1.512 (2)
N2—C3	1.358 (2)	C5—H5A	0.9700
N2—H2A	0.86 (2)	C5—H5B	0.9700
N2—H2B	0.88 (2)	C7—C8	1.384 (2)
N3—C4	1.324 (2)	C8—C9	1.376 (3)
N3—C1	1.358 (2)	C8—H8	0.9300
N4—C4	1.328 (2)	C9—C10	1.365 (3)
N4—C3	1.3570 (19)	C9—H9	0.9300
N5—C6	1.354 (2)	C10—C11	1.368 (3)
N5—C7	1.400 (2)	C10—H10	0.9300
N5—H5	0.856 (19)	C11—H11	0.9300
			
C4—S1—C5	102.83 (8)	C6—C5—S1	111.72 (12)
C1—N1—H1A	118.3 (14)	C6—C5—H5A	109.3
C1—N1—H1B	117.3 (15)	S1—C5—H5A	109.3
H1A—N1—H1B	124 (2)	C6—C5—H5B	109.3
C3—N2—H2A	117.2 (13)	S1—C5—H5B	109.3
C3—N2—H2B	117.6 (15)	H5A—C5—H5B	107.9
H2A—N2—H2B	110 (2)	O1—C6—N5	124.47 (16)
C4—N3—C1	114.94 (13)	O1—C6—C5	121.07 (15)
C4—N4—C3	115.04 (13)	N5—C6—C5	114.46 (14)
C6—N5—C7	129.23 (14)	N6—C7—C8	123.05 (16)
C6—N5—H5	114.6 (12)	N6—C7—N5	112.92 (13)
C7—N5—H5	116.2 (12)	C8—C7—N5	124.02 (15)
C7—N6—C11	116.91 (15)	C9—C8—C7	118.03 (18)
N1—C1—N3	115.65 (15)	C9—C8—H8	121.0
N1—C1—C2	122.72 (15)	C7—C8—H8	121.0
N3—C1—C2	121.63 (15)	C10—C9—C8	120.00 (18)
C1—C2—C3	117.79 (14)	C10—C9—H9	120.0
C1—C2—H2	121.1	C8—C9—H9	120.0
C3—C2—H2	121.1	C9—C10—C11	117.86 (18)
N4—C3—N2	116.08 (15)	C9—C10—H10	121.1
N4—C3—C2	121.51 (14)	C11—C10—H10	121.1
N2—C3—C2	122.39 (15)	N6—C11—C10	124.13 (18)
N3—C4—N4	128.88 (14)	N6—C11—H11	117.9
N3—C4—S1	119.16 (12)	C10—C11—H11	117.9
N4—C4—S1	111.95 (12)		
			
C4—N3—C1—N1	−174.86 (14)	C7—N5—C6—O1	−0.5 (3)
C4—N3—C1—C2	5.2 (2)	C7—N5—C6—C5	179.12 (15)
N1—C1—C2—C3	175.39 (15)	S1—C5—C6—O1	105.06 (17)
N3—C1—C2—C3	−4.7 (2)	S1—C5—C6—N5	−74.58 (17)
C4—N4—C3—N2	−176.87 (14)	C11—N6—C7—C8	1.2 (2)
C4—N4—C3—C2	1.5 (2)	C11—N6—C7—N5	−178.11 (15)
C1—C2—C3—N4	1.2 (2)	C6—N5—C7—N6	−178.11 (16)
C1—C2—C3—N2	179.38 (15)	C6—N5—C7—C8	2.5 (3)
C1—N3—C4—N4	−2.5 (2)	N6—C7—C8—C9	−1.7 (3)
C1—N3—C4—S1	177.30 (10)	N5—C7—C8—C9	177.57 (17)
C3—N4—C4—N3	−0.8 (2)	C7—C8—C9—C10	0.7 (3)
C3—N4—C4—S1	179.39 (10)	C8—C9—C10—C11	0.7 (3)
C5—S1—C4—N3	3.32 (14)	C7—N6—C11—C10	0.2 (3)
C5—S1—C4—N4	−176.85 (11)	C9—C10—C11—N6	−1.2 (3)
C4—S1—C5—C6	87.65 (13)		

### 2-[(4,6-Diaminopyrimidin-2-yl)sulfanyl]-N-(pyridin-2-yl)acetamide (I) Hydrogen-bond geometry (Å, º)

**Table:**

*D*—H···*A*	*D*—H	H···*A*	*D*···*A*	*D*—H···*A*
N5—H5···N3	0.86 (2)	2.18 (2)	2.975 (2)	154 (2)
C8—H8···O1	0.93	2.31	2.894 (2)	121
N2—H2*B*···N4^i^	0.88 (2)	2.20 (2)	3.082 (2)	178 (2)
N1—H1*A*···N6^ii^	0.86 (2)	2.38 (2)	3.174 (2)	155 (2)
N2—H2*A*···O1^iii^	0.86 (2)	2.13 (2)	2.956 (2)	159 (2)

Symmetry codes: (i) −*x*+2, −*y*+1, −*z*+2; (ii) −*x*+1, −*y*+1, −*z*+1; (iii) *x*+1, *y*+1, *z*.

### 2-[(4,6-Diaminopyrimidin-2-yl)sulfanyl]-N-(pyrazin-2-yl)acetamide (II) Crystal data

**Table:**

C_10_H_11_N_7_OS	*F*(000) = 576
*M**_r_* = 277.32	*D*_x_ = 1.453 Mg m^−^^3^
Monoclinic, *P*2_1_/*n*	Mo *K*α radiation, λ = 0.71073 Å
*a* = 12.1333 (5) Å	Cell parameters from 3124 reflections
*b* = 8.1561 (3) Å	θ = 2.2–28.3°
*c* = 12.8442 (5) Å	µ = 0.26 mm^−^^1^
β = 94.307 (3)°	*T* = 293 K
*V* = 1267.48 (9) Å^3^	Block, yellow
*Z* = 4	0.28 × 0.25 × 0.20 mm

### 2-[(4,6-Diaminopyrimidin-2-yl)sulfanyl]-N-(pyrazin-2-yl)acetamide (II) Data collection

**Table:**

Bruker SMART APEXII area-detector diffractometer	1320 reflections with *I* > 2σ(*I*)
Radiation source: X-ray	*R*_int_ = 0.084
ω and φ scans	θ_max_ = 28.3°, θ_min_ = 2.2°
Absorption correction: multi-scan (*SADABS*; Bruker, 2008)	*h* = −16→12
*T*_min_ = 0.723, *T*_max_ = 0.863	*k* = −10→9
11968 measured reflections	*l* = −17→17
3124 independent reflections	

### 2-[(4,6-Diaminopyrimidin-2-yl)sulfanyl]-N-(pyrazin-2-yl)acetamide (II) Refinement

**Table:**

Refinement on *F*^2^	Primary atom site location: structure-invariant direct methods
Least-squares matrix: full	Secondary atom site location: difference Fourier map
*R*[*F*^2^ > 2σ(*F*^2^)] = 0.054	Hydrogen site location: mixed
*wR*(*F*^2^) = 0.126	H atoms treated by a mixture of independent and constrained refinement
*S* = 0.94	*w* = 1/[σ^2^(*F*_o_^2^) + (0.0452*P*)^2^] where *P* = (*F*_o_^2^ + 2*F*_c_^2^)/3
3124 reflections	(Δ/σ)_max_ = 0.001
192 parameters	Δρ_max_ = 0.20 e Å^−^^3^
0 restraints	Δρ_min_ = −0.23 e Å^−^^3^

### 2-[(4,6-Diaminopyrimidin-2-yl)sulfanyl]-N-(pyrazin-2-yl)acetamide (II) Special details

**Table:**

Geometry. All esds (except the esd in the dihedral angle between two l.s. planes) are estimated using the full covariance matrix. The cell esds are taken into account individually in the estimation of esds in distances, angles and torsion angles; correlations between esds in cell parameters are only used when they are defined by crystal symmetry. An approximate (isotropic) treatment of cell esds is used for estimating esds involving l.s. planes.

### 2-[(4,6-Diaminopyrimidin-2-yl)sulfanyl]-N-(pyrazin-2-yl)acetamide (II) Fractional atomic coordinates and isotropic or equivalent isotropic displacement parameters (Å^2^)

**Table:**

	*x*	*y*	*z*	*U*_iso_*/*U*_eq_	
S1	0.77107 (6)	0.20394 (11)	0.50448 (6)	0.0525 (3)	
O1	1.06221 (18)	0.2503 (3)	0.55054 (17)	0.0779 (8)	
N1	0.6951 (3)	0.2961 (4)	0.8740 (2)	0.0651 (9)	
H1A	0.651 (3)	0.297 (4)	0.923 (3)	0.084 (13)*	
H1B	0.760 (3)	0.342 (4)	0.881 (3)	0.079 (13)*	
N2	0.4369 (2)	0.0046 (4)	0.6423 (3)	0.0635 (9)	
H2A	0.391 (2)	−0.015 (4)	0.687 (2)	0.060 (11)*	
H2B	0.423 (2)	−0.044 (4)	0.571 (3)	0.080 (11)*	
N3	0.72886 (19)	0.2473 (3)	0.70403 (17)	0.0452 (7)	
N4	0.59901 (19)	0.1011 (3)	0.58901 (17)	0.0463 (7)	
N5	0.9712 (2)	0.1889 (3)	0.69294 (19)	0.0494 (7)	
H5	0.912 (2)	0.197 (4)	0.719 (2)	0.057 (11)*	
N6	1.0164 (2)	0.0445 (3)	0.84291 (19)	0.0545 (7)	
N7	1.2266 (2)	−0.0273 (4)	0.7815 (2)	0.0659 (8)	
C1	0.6580 (3)	0.2321 (4)	0.7813 (2)	0.0450 (8)	
C2	0.5579 (2)	0.1540 (4)	0.7649 (2)	0.0466 (8)	
H2	0.509877	0.146453	0.817830	0.056*	
C3	0.5306 (2)	0.0872 (4)	0.6680 (2)	0.0438 (8)	
C4	0.6909 (2)	0.1845 (4)	0.6132 (2)	0.0421 (7)	
C5	0.8779 (2)	0.3427 (4)	0.5525 (2)	0.0497 (9)	
H5A	0.897729	0.412594	0.495784	0.060*	
H5B	0.849528	0.412281	0.605640	0.060*	
C6	0.9796 (3)	0.2561 (4)	0.5981 (2)	0.0499 (9)	
C7	1.0498 (2)	0.0968 (4)	0.7528 (2)	0.0440 (8)	
C8	1.1544 (3)	0.0605 (4)	0.7222 (3)	0.0613 (10)	
H8	1.174535	0.098921	0.658172	0.074*	
C9	1.1922 (3)	−0.0808 (4)	0.8710 (3)	0.0610 (10)	
H9	1.239366	−0.144894	0.914481	0.073*	
C10	1.0895 (3)	−0.0446 (4)	0.9011 (2)	0.0598 (9)	
H10	1.069630	−0.083866	0.965011	0.072*	

### 2-[(4,6-Diaminopyrimidin-2-yl)sulfanyl]-N-(pyrazin-2-yl)acetamide (II) Atomic displacement parameters (Å^2^)

**Table:**

	*U*^11^	*U*^22^	*U*^33^	*U*^12^	*U*^13^	*U*^23^
S1	0.0478 (5)	0.0731 (6)	0.0376 (4)	−0.0116 (5)	0.0092 (3)	−0.0006 (4)
O1	0.0459 (15)	0.134 (3)	0.0572 (14)	0.0005 (14)	0.0236 (12)	0.0208 (14)
N1	0.062 (2)	0.091 (2)	0.0445 (17)	−0.021 (2)	0.0163 (16)	−0.0161 (17)
N2	0.0446 (19)	0.090 (3)	0.0569 (19)	−0.0240 (17)	0.0124 (16)	−0.0020 (18)
N3	0.0437 (16)	0.0552 (19)	0.0379 (13)	−0.0049 (12)	0.0104 (12)	−0.0024 (12)
N4	0.0372 (15)	0.0617 (18)	0.0408 (14)	−0.0088 (14)	0.0095 (12)	−0.0002 (13)
N5	0.0379 (18)	0.069 (2)	0.0428 (15)	−0.0018 (16)	0.0162 (13)	0.0043 (14)
N6	0.0478 (17)	0.072 (2)	0.0447 (15)	0.0030 (15)	0.0119 (13)	0.0055 (14)
N7	0.0512 (19)	0.079 (2)	0.0690 (19)	0.0126 (17)	0.0169 (15)	0.0064 (17)
C1	0.049 (2)	0.046 (2)	0.0402 (16)	0.0041 (16)	0.0087 (15)	−0.0021 (15)
C2	0.042 (2)	0.058 (2)	0.0416 (17)	−0.0005 (17)	0.0110 (14)	0.0027 (15)
C3	0.0338 (19)	0.049 (2)	0.0493 (18)	0.0015 (16)	0.0076 (15)	0.0061 (16)
C4	0.0395 (19)	0.047 (2)	0.0410 (16)	0.0036 (16)	0.0083 (14)	0.0024 (15)
C5	0.047 (2)	0.055 (2)	0.0479 (18)	−0.0136 (16)	0.0103 (15)	0.0067 (15)
C6	0.044 (2)	0.061 (2)	0.0449 (18)	−0.0142 (17)	0.0067 (16)	0.0004 (16)
C7	0.0338 (19)	0.053 (2)	0.0463 (18)	−0.0016 (16)	0.0105 (15)	−0.0048 (16)
C8	0.053 (2)	0.076 (3)	0.057 (2)	0.007 (2)	0.0187 (18)	0.0088 (19)
C9	0.054 (2)	0.073 (3)	0.057 (2)	0.012 (2)	0.0051 (17)	0.0020 (19)
C10	0.061 (3)	0.069 (3)	0.0508 (19)	0.002 (2)	0.0107 (18)	0.0063 (19)

### 2-[(4,6-Diaminopyrimidin-2-yl)sulfanyl]-N-(pyrazin-2-yl)acetamide (II) Geometric parameters (Å, º)

**Table:**

S1—C4	1.768 (3)	N6—C7	1.325 (3)
S1—C5	1.795 (3)	N6—C10	1.331 (4)
O1—C6	1.213 (3)	N7—C8	1.326 (4)
N1—C1	1.346 (4)	N7—C9	1.326 (4)
N1—H1A	0.86 (3)	C1—C2	1.374 (4)
N1—H1B	0.88 (3)	C2—C3	1.375 (4)
N2—C3	1.341 (4)	C2—H2	0.9300
N2—H2A	0.85 (3)	C5—C6	1.502 (4)
N2—H2B	1.00 (3)	C5—H5A	0.9700
N3—C4	1.325 (3)	C5—H5B	0.9700
N3—C1	1.367 (3)	C7—C8	1.389 (4)
N4—C4	1.323 (3)	C8—H8	0.9300
N4—C3	1.364 (3)	C9—C10	1.364 (4)
N5—C6	1.347 (4)	C9—H9	0.9300
N5—C7	1.398 (4)	C10—H10	0.9300
N5—H5	0.82 (3)		
			
C4—S1—C5	102.17 (14)	N4—C4—S1	111.4 (2)
C1—N1—H1A	118 (2)	N3—C4—S1	119.0 (2)
C1—N1—H1B	120 (2)	C6—C5—S1	112.8 (2)
H1A—N1—H1B	122 (3)	C6—C5—H5A	109.0
C3—N2—H2A	121 (2)	S1—C5—H5A	109.0
C3—N2—H2B	120.4 (17)	C6—C5—H5B	109.0
H2A—N2—H2B	118 (3)	S1—C5—H5B	109.0
C4—N3—C1	114.1 (2)	H5A—C5—H5B	107.8
C4—N4—C3	114.7 (3)	O1—C6—N5	124.1 (3)
C6—N5—C7	128.4 (3)	O1—C6—C5	120.5 (3)
C6—N5—H5	118 (2)	N5—C6—C5	115.4 (3)
C7—N5—H5	114 (2)	N6—C7—C8	121.7 (3)
C7—N6—C10	115.5 (3)	N6—C7—N5	114.4 (3)
C8—N7—C9	116.0 (3)	C8—C7—N5	124.0 (3)
N1—C1—N3	114.9 (3)	N7—C8—C7	122.1 (3)
N1—C1—C2	123.3 (3)	N7—C8—H8	119.0
N3—C1—C2	121.9 (3)	C7—C8—H8	119.0
C1—C2—C3	118.2 (3)	N7—C9—C10	121.9 (3)
C1—C2—H2	120.9	N7—C9—H9	119.1
C3—C2—H2	120.9	C10—C9—H9	119.1
N2—C3—N4	114.2 (3)	N6—C10—C9	122.9 (3)
N2—C3—C2	124.3 (3)	N6—C10—H10	118.5
N4—C3—C2	121.4 (3)	C9—C10—H10	118.5
N4—C4—N3	129.6 (3)		
			
C4—N3—C1—N1	−179.7 (3)	C7—N5—C6—O1	−2.8 (5)
C4—N3—C1—C2	1.7 (4)	C7—N5—C6—C5	177.6 (3)
N1—C1—C2—C3	−177.3 (3)	S1—C5—C6—O1	105.2 (3)
N3—C1—C2—C3	1.3 (5)	S1—C5—C6—N5	−75.2 (3)
C4—N4—C3—N2	179.0 (3)	C10—N6—C7—C8	−0.1 (5)
C4—N4—C3—C2	−0.9 (4)	C10—N6—C7—N5	179.5 (3)
C1—C2—C3—N2	178.4 (3)	C6—N5—C7—N6	−178.8 (3)
C1—C2—C3—N4	−1.7 (5)	C6—N5—C7—C8	0.8 (5)
C3—N4—C4—N3	4.6 (5)	C9—N7—C8—C7	1.1 (5)
C3—N4—C4—S1	−177.3 (2)	N6—C7—C8—N7	−0.4 (5)
C1—N3—C4—N4	−5.0 (5)	N5—C7—C8—N7	180.0 (3)
C1—N3—C4—S1	177.1 (2)	C8—N7—C9—C10	−1.3 (5)
C5—S1—C4—N4	172.4 (2)	C7—N6—C10—C9	−0.1 (5)
C5—S1—C4—N3	−9.3 (3)	N7—C9—C10—N6	0.9 (5)
C4—S1—C5—C6	93.4 (2)		

### 2-[(4,6-Diaminopyrimidin-2-yl)sulfanyl]-N-(pyrazin-2-yl)acetamide (II) Hydrogen-bond geometry (Å, º)

**Table:**

*D*—H···*A*	*D*—H	H···*A*	*D*···*A*	*D*—H···*A*
N5—H5···N3	0.82 (3)	2.25 (3)	2.993 (4)	151 (3)
C8—H8···O1	0.93	2.24	2.854 (4)	123
N2—H2*B*···N4^i^	1.00 (3)	2.11 (3)	3.092 (4)	169 (3)
N1—H1*A*···O1^ii^	0.86 (3)	2.06 (4)	2.904 (4)	167 (3)
N2—H2*A*···N7^iii^	0.85 (3)	2.41 (3)	3.235 (4)	164 (3)
C9—H9···O1^iv^	0.93	2.56	3.368 (4)	145

Symmetry codes: (i) −*x*+1, −*y*, −*z*+1; (ii) *x*−1/2, −*y*+1/2, *z*+1/2; (iii) *x*−1, *y*, *z*; (iv) −*x*+5/2, *y*−1/2, −*z*+3/2.
